# DkWRKY transcription factors enhance persimmon resistance to *Colletotrichum horii* by promoting lignin accumulation through *DkCAD1* promotor interaction

**DOI:** 10.1007/s44154-024-00154-0

**Published:** 2024-02-26

**Authors:** Hanyue Fan, Xiaoxia Shen, Yu Ding, Yongkuan Li, Shuyuan Liu, Yong Yang, Yuduan Ding, Changfei Guan

**Affiliations:** State Key Laboratory of Crop Stress Biology for Arid Areas, College of Horticulture, Northwest A&F University, Yangling, Shaanxi China

**Keywords:** Persimmon, *DkCAD1*, *DkWRKY*, Lignin, *Colletotrichum horii*, SA, JA

## Abstract

Persimmon anthracnose, a severe disease caused by the hemibiotrophic fungus *Colletotrichum horii*, poses a substantial threat to China’s persimmon industry. Previous research showed that ‘Kangbing Jianshi’ cultivar exhibits strong resistance to anthracnose. Notably, ‘Kangbing Jianshi’ branches exhibit greater lignification compared with the susceptible ‘Fuping Jianshi’ cultivar. In this study, higher lignin content was observed in ‘Kangbing Jianshi’ compared with ‘Fuping Jianshi’, and this difference was associated with disease resistance. Transcriptome and metabolome analyses revealed that the majority of differentially expressed genes and differentially accumulated metabolites were primarily enriched in the phenylpropanoid biosynthesis and lignin synthesis pathways. Furthermore, significant upregulation of *DkCAD1*, a pivotal gene involved in lignin metabolism, was observed in the resistant cultivar when inoculated with *C. horii*. Transient overexpression of *DkCAD1* substantially increased lignin content and improved resistance to *C. horii* in a susceptible cultivar. Furthermore, through yeast one-hybrid (Y1H) assays, we identified two WRKY transcription factors, DkWRKY8 and DkWRKY10, which interacts with the *DkCAD1* promoter and induces its activity. Overexpression of *DkWRKY8* and *DkWRKY10* not only increased leaf lignin content but also enhanced persimmon tolerance to *C*. *horii*. Moreover, the expression levels of *DkCAD1*, *DkWRKY8*, and *DkWRKY10* were significantly increased in response to salicylic acid and jasmonic acid in the resistant cultivar. These findings enhance our understanding of the molecular functions of *DkWRKY8*, *DkWRKY10*, and *DkCAD1* in persimmons, as well as their involvement in molecular breeding processes in persimmons.

**Supplementary Information:**

The online version contains supplementary material available at 10.1007/s44154-024-00154-0.

## Introduction

Persimmon (*Diospyros kaki* Thunb.), a member of the *Diospyros* genus, is a prominent commercial fruit crop of great importance. It is primarily cultivated in East Asian countries, with notable production in China, Japan, and Korea. (Luo and Wang 2008; Yamada and Sato 2016). With the expansion of persimmon cultivation, germplasm collection and analysis become increasingly important. This effort preserve genetic diversity and aids in screening and breeding for superior disease-resistant persimmon varieties (Greene and Morris 2001). The National Field Genebank for Persimmon in the Yangling District of Shaanxi Province has amassed over 1000 persimmon germplasm and varieties, exhibiting substantial genetic diversity from various regions in China and worldwide (Guan et al. 2019; Guan et al. 2020b). In a prior study, we evaluated 142 persimmon germplasms for their response to *Colletotrichum horii*, revealing notable variations among the examined accessions, with the most displaying high susceptibility and a few exhibiting hypersensitive reactions (Guan et al. 2022).

Persimmon anthracnose is a severe disease that affects prominent persimmon cultivation regions. It is caused by the pathogen *C. horii*, which was renamed to distinguish it from the broader *C. gloeosporioides* species complex (Weir and Johnston 2010). *C. horii* is highly destructive to persimmons plants, leading to leaf defoliation, fruit decay, and even plant demise (Zhang and Xu 2005). This disease has become endemic in certain Chinese regions, leading to substantial losses in both quality and production (Deng et al. 2019).

Lignin, a key component of plant cell walls, serves as the initial physical barrier against pathogen infection by inducing cell walls lignification, which protects neighboring plant cells from further damage (Khasin et al. 2021; Quiroz-Figueroa et al. 2023). Cinnamyl alcohol dehydrogenase (CAD) is a crucial enzyme in lignin biosynthesis. Research has identified key lignin synthesis enzymes (*AtCAD1*, *AtCAD4,* and *AtCAD5*) in *Arabidopsis* (Sibout et al. 2003; Kim et al. 2004; Eudes et al. 2006). In rice, the relationship between *OsCAD2* and *OsCAD7* is associated with the lignin content, with *OsCAD2* primarily responsible for the biosynthesis of monolignols in rice lignin among the *OsCAD* genes (Li et al. 2009; Hirano et al. 2012). The CAD7 subfamily protein has been identified as a negative regulator, promoting host plant infection by suppressing immunity factors, including callose deposition and plant reactive oxygen species (ROS) burst (Li et al. 2019). These findings highlight the crucial role of the *CAD* gene in lignin synthesis and disease resistance in persimmon. However, studies on CAD protein-mediated resistance to anthracnose remain limited.

WRKY transcription factors are crucial in regulating disease resistance across various hosts, including *Arabidopsis* (Birkenbihl et al. 2018), rice (Li et al. 2021), cucumber (Luan et al. 2019), pepper (Hussain et al. 2019) and grape plants (Wang et al. 2020). In recent years, evidence has increasingly linked WRKY transcription factors to the signaling pathways of salicylic acid (SA) and jasmonic acid (JA) in plant defense responses. For instance, overexpression *CmWRKY15-1* in chrysanthemums infected with *Puccinia horiana* increased endogenous SA level and an up-regulated SA synthesis pathway genes (Bi et al. 2021). Additionally, exogenous SA and JA treatments significantly induced the *NtWRKY50* expression, enhancing resistance against *Ralstonia solanacearum* (Liu et al. 2017). Furthermore, WRKY transcription factors initiate the plant immune response by binding to cis-acting elements in specific genes promoter (Eulgem and Somssich 2007). For example, AtWRKY57 directly binds to the promoters of *JAZ1* and *JAZ5*, suppressing the JA signal transduction pathway and negatively regulating *Botrytis cinerea* resistance in *Arabidopsis* (Jiang and Yu 2016). In pepper, CaWRKY6 enhances resistance against *R. solanacearum* by interacting with the *CaWRKY40* promoter (Cai et al. 2015). Notably, WRKY transcription factors play crucial biological roles in interactions among economically significant crops. For instance, banana MaWRKY1 and MaWRKY2 induce resistance against *C. musae* by binding to pathogenesis-related genes promoters (Shan et al. 2016); In apples, overexpressing *MdWRKY100* enhances resistance against to *C. gloeosporioides* (Zhang et al. 2019); Conversely, *HbWRKY40* from *Hevea brasiliensis* triggers a burst of ROS in tobacco, increasing disease resistance in *Arabidopsis* (Yang et al. 2020); Additionally, upregulating *JrWRKY21* in walnut positively regulates its resistance to *C. gloeosporioides* (Zhou et al. 2022). Despite these findings in various plant species, the potential role of WRKY transcription factors in persimmon disease resistance remains largely unexplored. Finally, both JA and SA are resistance hormones that activate plant defense against insect attack and necrotrophic pathogens. JA primarily regulates disease resistance against necrotrophic pathogens, whereas SA regulates broad-spectrum resistance against hemibiotrophic and biotrophic pathogens (Fu et al. 2012). These hormones typically work antagonistically, with SA and JA associated primarily with biotrophic resistance and necrotrophic resistance, respectively (Xie et al. 2022). Surprisingly, no studies have investigated the roles of SA and JA in persimmon anthracnose disease resistance.

In the present study, we conducted a comparative analysis of disease progression, lignin accumulation, and *DkCAD1* gene expression (a lignin metabolism-related gene) in branches of the susceptible cultivar ‘Fuping Jianshi’ (S var.) and the resistance cultivar ‘Kangbing Jianshi’ (R var.) after *C. horii* inoculation. Our findings suggest that lignin may play a key role in conferring the robust disease resistance observed in R var. We preliminary verified the biological function of *DkCAD1* in conferring resistance to *C. horii* in various persimmon varieties using a transient overexpression system in persimmon leaves. Additionally, two WRKY transcription factors, DkWRKY8 and DkWRKY10, were found to transactivate the *DkCAD1* promoter upon *C*. *horii* induction. This study elucidated variations in lignin content and JA and SA levels in resistant and susceptible persimmon cultivars and demonstrated the positive contributions of *DkCAD1*, *DkWRKY8*, and *DkWRKY10* to anthracnose resistance in persimmon.

## Results

### Morphological characteristics of persimmon anthracnose *C. horii* in various persimmon tissues

**Fig. 1:**
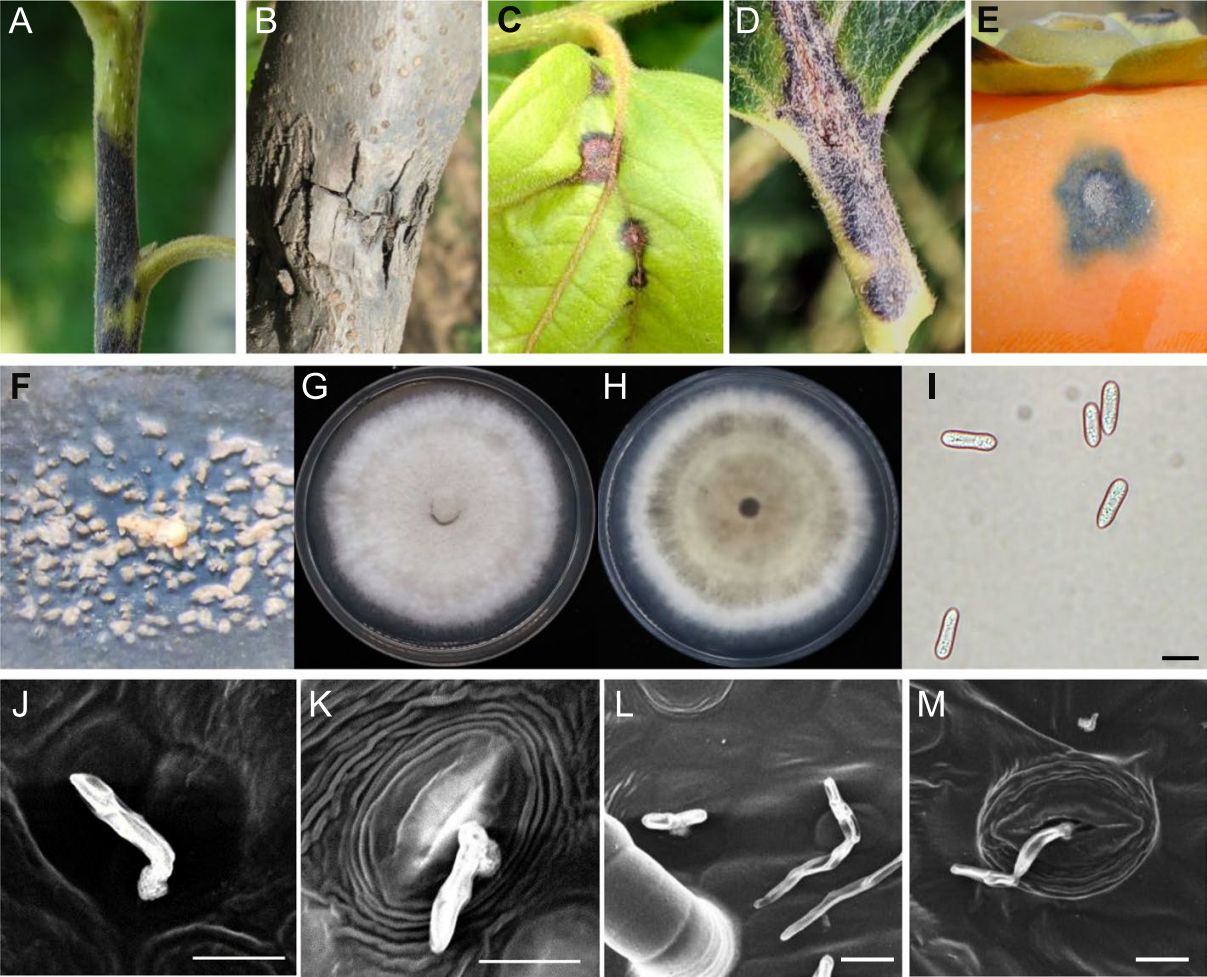
Morphological characteristics of persimmon anthracnose *C. horii* in various persimmon tissues. **A**-**E** Morphological characteristics of persimmon anthracnose *C. horii* in the new shoot (**A**), perennial branch (**B**), leaf (**C**), petiole (**D**), and fruit (**E**). **F** Stereo structure of *C. horii* conidia on the fruit surface of the ‘Fuping Jianzhi’ variety, as observed under the anatomical microscope. **G** and (**H**) Images from above (**G**) and below (**H**) of *C. horii* anthracnose cultured on the PDA plate for 10 days. **I** Conidia. **J**-**M** Microscopic view of *C. horii* invading the back of ‘Fuping Jianzhi’ leaves under scanning electron microscope. Scale bar = 10 μm

*C. horii* demonstrated the ability to infect various parts of the persimmon tree, including the trunk, branches, leaves, and fruit. Field observations revealed slight variations in disease symptoms across affected tissues. Afflicted branches exhibited the small black spots that progressively deepened into larger depressions. Over time, these sunken areas expanded, causing the bark to crack (Fig. 1A, B). On leaves, disease spots extended along the petiole and veins, leading to the formation of pink conidia (Fig. 1C, D). In fruit, affected areas exhibited depressions that evolved into soft black patches (Fig. 1E). In advanced fruit infections, substantial conidia masses became visible in the infected regions (Fig. 1F). The morphological characteristics of *C. horii* conidia from the diseased spots closely resembled those cultured on PDA plate (Fig. 1G, H), as previously described (Xie et al. 2010; Deng et al. 2019). Scanning electron microscopy revealed that *C. horii* anthracnose filtrates persimmon leaves via the elytra structure at the apex of the embryonic tube following conidia germination. Both infection structures of *C. horii* entered persimmon leaves through intercellular spaces and stomata, indicating multiple pathways for *C. horii* infections (Fig. 1I–M).

### Characteristics and physiological changes in persimmon anthracnose in two persimmon cultivars

**Fig. 2:**
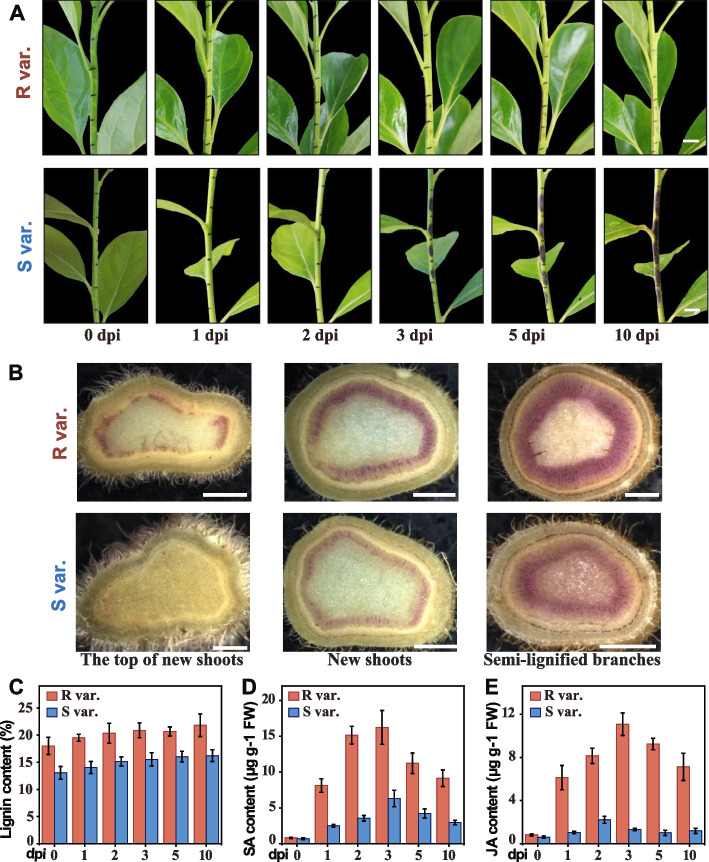
Characteristics and physiological changes in persimmon anthracnose in two persimmon cultivars. **A** Characteristic of the highly resistant cultivar ‘Kangbing Jianshi’ (R var.) and the highly susceptible cultivar ‘Fuping Jianshi’ (S var.) following inoculation with *C. horii*. Images were taken at 0, 1-, 2-, 3-, 5-, 10-days post-inoculation (dpi). Scale bars = 1 cm. **B** Lignin staining on the cross sections of R var. and S var. branches. Samples were collected from the apical region of young shoots, young shoots, and semi-lignified branches. Scale bars = 10 µm. **C** Lignin content in branches of R var. and S var. cultivars following inoculation with *C. horii*. **D** Endogenous salicylic acid (SA) and (**E**) jasmonic acid (JA) contents in R var. and S var. Error bars represent standard deviation (*n* = 3)

Significant differences in anthracnose symptoms were observed between the highly susceptible and highly resistant cultivars upon field inoculation with *C. horii*. At 3 days post inoculation (dpi), distinct black spots appeared on the branches of S var. (‘Fuping Jianshi’). As the disease progressed, these lesions rapidly expanded outward, connecting with adjacent lesions, ultimately infecting the entire branch and producing conidia by 10 dpi. In contrast, the branches of the R var. (‘Kangbing Jianshi’) showed only mild symptoms after 5 dpi, characterized by small grayish spots in the early stages of disease progression. Lesions in R var. increased minimally over time, with no conidia observed (Fig. 2A). To explore the relationship between lignin and persimmon disease resistance, we determined the lignin content in various resistant cultivars using Klason method, a reliable technique for isolating lignin fractions (Abdelrahman and Galiwango 2018). Results indicated higher lignin content in R var. compared with S var. Following *C. horii* inoculation, R var. displayed a noticeable increase in lignin accumulation, whereas no significant change was observed in S var., suggesting a potential role of lignin in disease resistance (Fig. 2B). Using phloroglucinol, lignin content in branches of different cultivars was assessed at various growth stages, revealing early lignin deposition in apical regions of young shoots in resistant cultivars, whereas susceptible cultivars showed minimal lignin expression at the same growth stage. The depth of lignin staining significantly increased in the semi-lignified branches of both cultivars, with a more pronounced increase observed in R var. compared with S var. (Fig. 2C).

SA and JA are pivotal hormones in plant disease resistance. To investigate their induction after pathogen infection, we measured SA and JA levels in R var. and S var. at various stages of *C. horii* disease progression. After pathogen infection, SA levels in R var. exhibited rapid elevation, peaking at 3 dpi, and gradually decreasing at 5 dpi while remaining significantly higher than preinfection levels (Fig. 2D). Similarly, JA levels followed a comparable trend in R var., whereas no distinct pattern emerged in S var. (Fig. 2E). These findings suggest that JA levels in R var. aligned with SA trends, but such alignment was not evident in S var.

### Transcriptome and metabolome analysis in two persimmon cultivars infected by *C. horii*

**Fig. 3:**
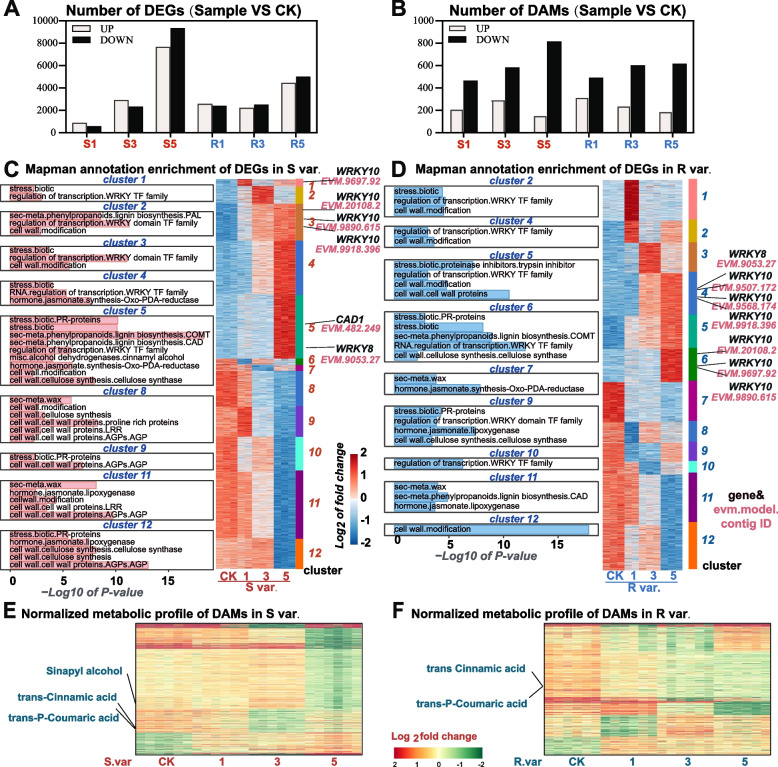
Transcriptome and metabolome analysis in ‘Fuping Jianshi’ (S var.) and ‘Kangbing Jianshi’ (R var.) infected by C. *horii.* **A**, **B** The number of DEGs and DAMs under control and *C. horii* treatment. The bar chart shows the numbers of DEGs and DAMs. DEG, differentially expressed genes. DAM, differentially accumulated metabolites. 1, 3, and 5 represent the time points for collecting samples. **C**, **D** Mapman function enrichment analysis of DEGs and DAMs in S var. and R var. The histograms illustrated the –log10 of *P*-value of each term of enrichment. **E**, **F** Heatmaps of Metabolic profiles of DAMs, which illustrated the log_2_fold change Normalized Metabolic value

To explore changes in persimmons genes associated with varying resistance levels during *C. horii* infection, we conducted a comprehensive analysis of the transcriptome and metabolome analysis of S var. (‘Fuping Jianshi’) and R var. (‘Kangbing Jianshi’) persimmons cultivars following inoculation with *C. horii* spore suspension. Branch samples were collected at different time points, including 1, 3, and 5 dpi, as well as an uninfected (CK), to identify differentially expressed genes (DEGs, Fig. 3A) and differentially accumulated metabolites (Fig. 3B). For transcriptome, we performed cluster analysis on the two groups of differential genes, and generated 12 groups of genes with different expression patterns (Fig. 3C, D). According to the clustering results, most of the genes in S var. were differentially expressed at 5 days, and more genes in R var. occurred at 1 and 3 days compared with S var., which is one of the reasons why R var. is more resistant to disease. As part of the metabolome analysis, certain metabolites associated with phenylpropanoid biosynthesis exhibited heightened expression levels during infection (Fig. 3E, F; Table S1. Matrix of normalized metabolic profile of DAMs).

We performed GO, KEGG and Mapman annotation system enrichment analyses for these DEGs (Table S2. Matrix of -log10 *p*-value from GO annotation enrichment analysis of DEGs; Table S3. Matrix of -log10 *p*-value from KEGG annotation enrichment analysis of DEGs; Table S4. Matrix of -log10 *p*-value from Mapman annotation enrichment analysis of DEGs). Enrichment results supported by the two ontology annotation systems were concentrated in the pathways related to cell wall synthesis, lignin synthesis, jasmonic acid, and bio-stress resistance, all of which are related to the process of cells resisting the invasion of external microorganisms (Fig. 3C, D; Figure S1). In R var., the GO enrichment analysis of cluster 1 and cluster 2 in which genes were early up-regulated suggested that the salicylic acid signaling pathway and the wax biosynthetic pathway were activated, and the enrichment analysis of Mapman suggested cell wall modification, biotic stress and WRKY TF-related pathways are activated. The expression trend of cluster 1 in S var. was the same as that of cluster 2 in R var., although the enrichment results suggested the activation of salicylic acid signaling, jasmonic acid response, WRKY transcription factor and biotic stress-related pathways.

### Analysis of genes and metabolites associated with SA, JA, and lignin synthesis

According to the enrichment analysis after gene clustering, salicylic acid and jasmonic acid were both involved in the activation of plant resistance. However, there is an antagonistic effect of two plant hormones when they participate in the pathway of plant resistance (Figure S2; Table S5). In the R var., the expression of salicylic acid-related genes exhibited a peculiar up-regulation at 1 day post infection, whereas the upregulation of jasmonic acid-related genes was only marginal. Among the genes upregulated by S var., the number of jasmonic acid-related genes was higher than that of salicylic acid-related genes. After 3-5 days of treatment, the number of upregulated genes associated with salicylic acid and jasmonic acid was approximately equivalent in R var., while the number of upregulated genes related to jasmonic acid was significantly more than those associated with salicylic acid in S var. after 3-5 days of treatment (Figure S2; Table S5). Therefore, we speculate that both salicylic acid and jasmonic acid-related pathways are activated in S var., but jasmonic acid is dominant, while Salicylic acid plays a role in the early reaction of R var.

**Fig. 4:**
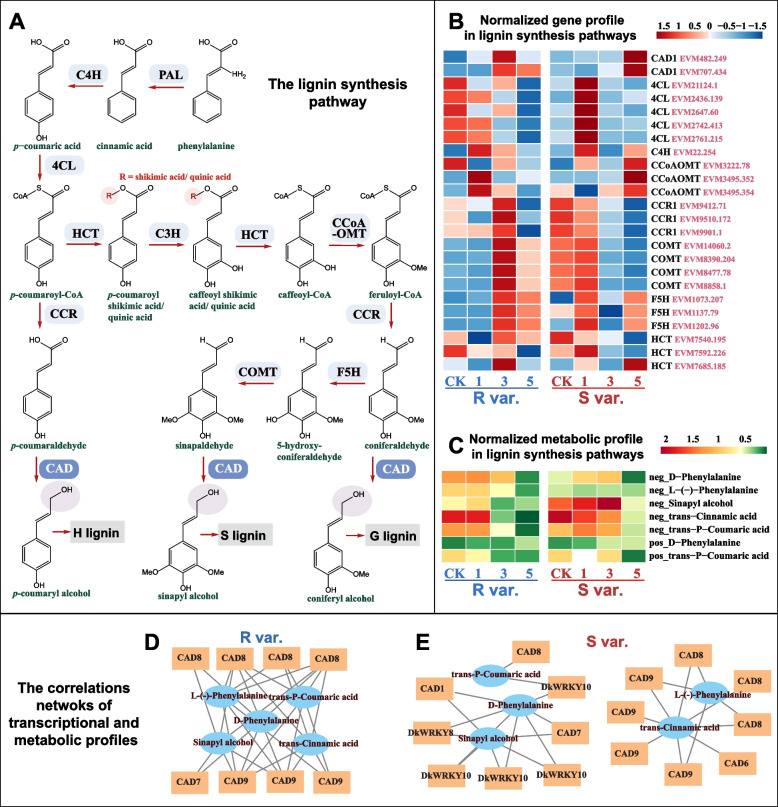
The lignin synthesis pathways and the expression level of related genes. **A** The simplified roadmap for the lignin synthesis pathway. **B** Gene expression pattern in lignin synthesis pathways from RNA-seq data (Normalized gene profile with Z-score value of Log2 (TPM+1)). **C** Metabolic profiling in lignin synthesis pathways (Normalized metabolite value was Log2fold change between metabolite content and detection-minimum). **D**, **E** The correlation networks in lignin synthesis pathways (Orange nodes represented DEGs in R var. or S var.; Blue nodes represented DAMs in R var. or S var.; Lines illustrated correlation relationship with Pearson coefficient > 0.8)

In the context of lignin synthesis (Fig. 4A), we examined transcriptome and metabolome profiles across different periods and varieties in lignin synthesis pathways (Fig. 4B, C). Notably, the different expression levels of *CAD1*, *4CL*, *CCoAOMT*, *CCR1*, *COMT*, *F5H*, and *HCT* related genes in resistant varieties compared with susceptible varieties (Fig. 4B; Table S6). CAD, a pivotal enzyme involved in the synthesis of three types of lignin, especially CAD1, exhibited higher expression during the early stages of infection. Moreover, the expression level of *CAD1* in resistant varieties was notably elevated before 3 dpi (Table S7). According to our omics data, the upregulation of DkCAD1 may contribute to increased lignin synthesis and enhanced persimmon resistance against *C. horii*. We also identified certain metabolites associated with lignin biosynthesis, which exhibited decreased expression levels during infection.

Correlation network analysis, integrating co-expression gene and metabolite profiling, highlighted the significant involvement of the lignin synthesis pathway. By calculating correlations between gene expression profiles and metabolites, we identified relationships with a Pearson correlation coefficient greater than 0.8 (Fig. 4D, E). Strong correlations were observed among five compounds involved in the lignin synthesis pathway [sinapyl alcohol, trans-cinnamic acid, trans-P-coumaric acid, D-phenylalanine and L-(-)-phenylalanine] and multiple *CAD* genes, as well as WRKY8 and WRKY10. In R var., five compounds exhibited significant correlations with the expression levels of *CAD7*, *CAD8*, and *CAD9*. In S var., L-(-)-phenylalanine and trans-cinnamic acid content was strongly associated with the expression of lignin synthesis genes *CAD6*, *CAD8*, and *CAD9*. Conversely, sinapyl alcohol, trans-P-coumaric acid and D-phenylalanine content was not only linked with the expression of lignin synthesis genes *CAD1*, *CAD7*, and *CAD8* but also robustly correlated with *WRKY8* and *WRKY10* expression. This suggests that WRKY8 and WRKY10 may play a role in regulating lignin synthesis in S var.

### Enhanced resistance to *C. horii *in S var. through *DkCAD1* overexpression promoting lignin accumulation

**Fig. 5:**
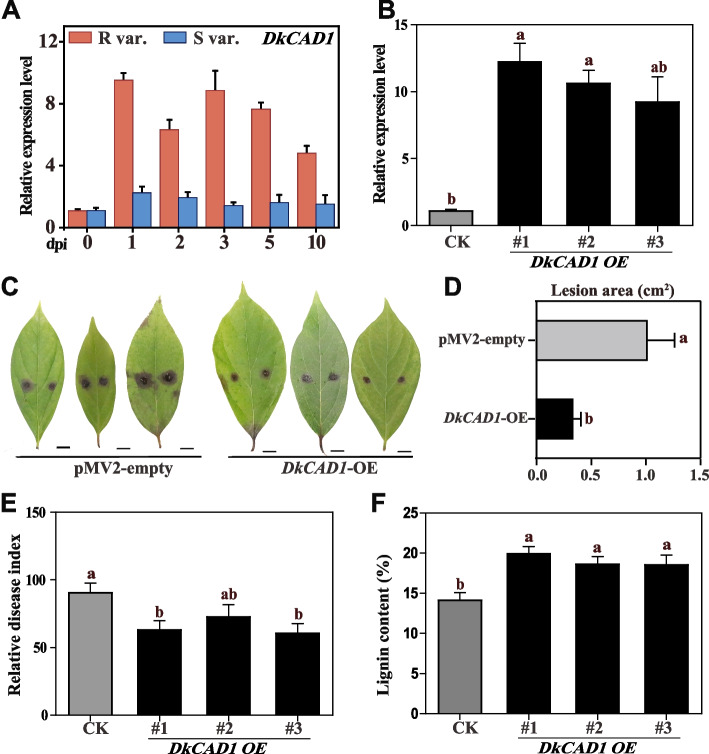
Enhanced resistance to *C. horii* in S var. through *DkCAD1* overexpression promoting lignin accumulation. **A** Relative expression of *DkCAD1* in inoculated persimmon branches. The samples were collected at 0, 1-, 2-, 3-, 5-, and 10-days post incubation (dpi). **B** Expression level of *DkCAD1* in persimmon leaves after transient overexpression of *DkCAD1* for 2 d. **C** Disease resistance of pMV-empty and *DkCAD1* overexpressing leaves. Scale bar = 1 cm. **D** Quantification of the data shown in (**A**), images were taken at 5 dpi. **E** Relative disease index in *DkCAD1*-overexpressing leaves after inoculation for 5 d. pMV2-GFP was used as a control. **F** The lignin content in *DkCAD1*-overexpressing leaves after inoculation for 5 d. Error bars indicate the standard deviation (*n*=3). The letters indicate significant differences according to one-way ANOVA (Tukey’s test; *p* < 0.05)

Further investigation was required to explore the relationship between lignin and disease resistance in persimmon. To do so, we isolated *DkCAD1*, a gene related to lignin metabolism. We observed that the expression of *DkCAD1* significantly increased in the branches of R var. infected by *C. horii* but remained unchanged in the branches of S var. (Fig. 5A). Specifically, at 1 dpi, the expression of *DkCAD1* in R var. increased by 9.5-fold compared with 0 dpi. The expression levels did not return to pre-infection levels even though they slightly declined after infection. To examine the role of *DkCAD1* in the plant–pathogen interaction, we used a transient overexpression system in persimmon leaves. As expected, we observed a substantial increase in *DkCAD1* transcription levels in S var. leaves after 2 days, with a >9-fold increase compared with the control, particularly in the overexpressed lines OE2, OE5, and OE6 (Fig. 5B). These results confirm the successful transformation of the *DkCAD1* gene into leaves. Subsequently, we inoculated the overexpressed lines OE2, OE5, and OE6 with *C. horii*. After 5 dpi, we observed a significant reduction in the lesion area and disease index in OE2 and OE6 throughout the study period (Fig. 5C–E). Additionally, the lignin content of the three groups was significantly higher compared with the control (Fig. 5F). These findings demonstrate that the overexpression of *DkCAD1* in persimmon effectively increases lignin content and enhances the resistance of S var. leaves against *C. horii*.

### Interaction between the *DkCAD1* promoter and DkWRKY8 and DkWRKY10

**Fig. 6:**
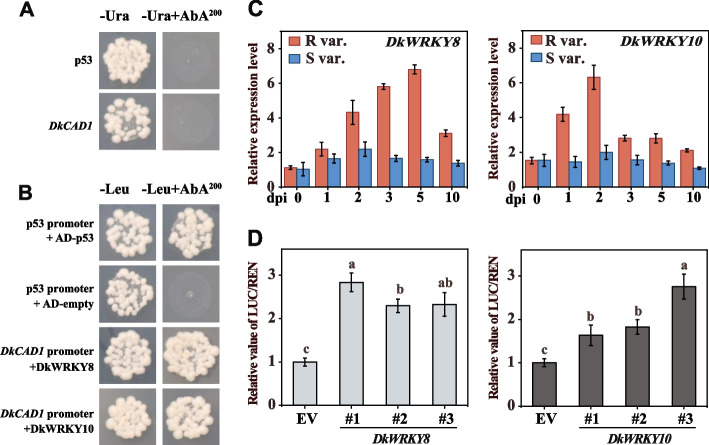
Interaction between the *DkCAD1* promoter and DkWRKY8 and DkWRKY10*.* **A** Self-activation of the *DkCAD1* promoter was detected on SD/−Ura medium with AbA (200 ng/mL). **B** Y1H assay of *DkCAD1* promoter with DkWRKY8 and DkWRKY10. The interaction was determined on the medium of SD/−Leu + AbA (200 ng/mL). **C** Relative expression of *DkWRKY8* and *DkWRKY10* in inoculated persimmon branches. The samples were collected at 0, 1-, 2-, 3-, 5-, and 10-days post incubation (dpi). **D** Ratios of LUC/REN on the promoter fragments of *DkCAD1* to DkWRKY8 and DkWRKY10. The LUC/REN ratio of the empty vector (EV) plus promoter was set as 1. Error bars represent the standard deviation (*n*=5). The letters indicate significant differences according to one-way ANOVA (Tukey’s test; *p* < 0.05)

To investigate the molecular mechanism underlying DkCAD1-mediated *C. horii* infection, we used the promoter of *DkCAD1* as a bait to screen for two WRKY transcription factors, DkWRKY8 and DkWRKY10. Y1H yeast cells containing the *DkCAD1* promoter fragment were unable to grow on SD/-Ura medium supplemented with 200 ng/ml Aureobasidin A (AbA). However, after transforming the PGADT7-*DkWRKY8* and PGADT7-*DkWRKY10* in Y1H yeast cells, they could grow on 200 ng/ml AbA on SD/-Leu medium, which indicated their binding to the promoter of *DkCAD1* (Fig. 6B, Figure S3). Subsequent, qRT-PCR results revealed significantly elevated expression levels of *DkWRKY8* and *DkWRKY10* in R var. compared with S var. The relative expression of *DkWRKY8* in R var. gradually increased, reaching its peak at 5 dpi, followed by a decrease at 10 dpi. Conversely, *DkWRKY10* was significantly up-regulated during the early stages of infection, peaking at 2 dpi, and then declining to a lower level but remaining higher than the 0-dpi level (Fig. 6C). To assess the impact of DkWRKY8 and DkWRKY10 on the transcriptional activity of the *DkCAD1* promoter, we conducted dual-luciferase assays. The results demonstrated that the LUC/REN values of *DkCAD1* promoter were significantly higher when cotransferred with DkWRKY8 and DkWRKY10 than the empty vector (EV) (Fig. 6D), suggesting the positive role of DkWRKY8 and DkWRKY10 in regulating the promoter activity of *DkCAD1*. These findings suggest that DkWRKY8 and DkWRKY10 can interact with the promoter of *DkCAD1* and positively regulate its activity.

### Enhanced resistance to *C. horii* in persimmon through *DkWRKY8* and *DkWRKY10* overexpression

**Fig. 7:**
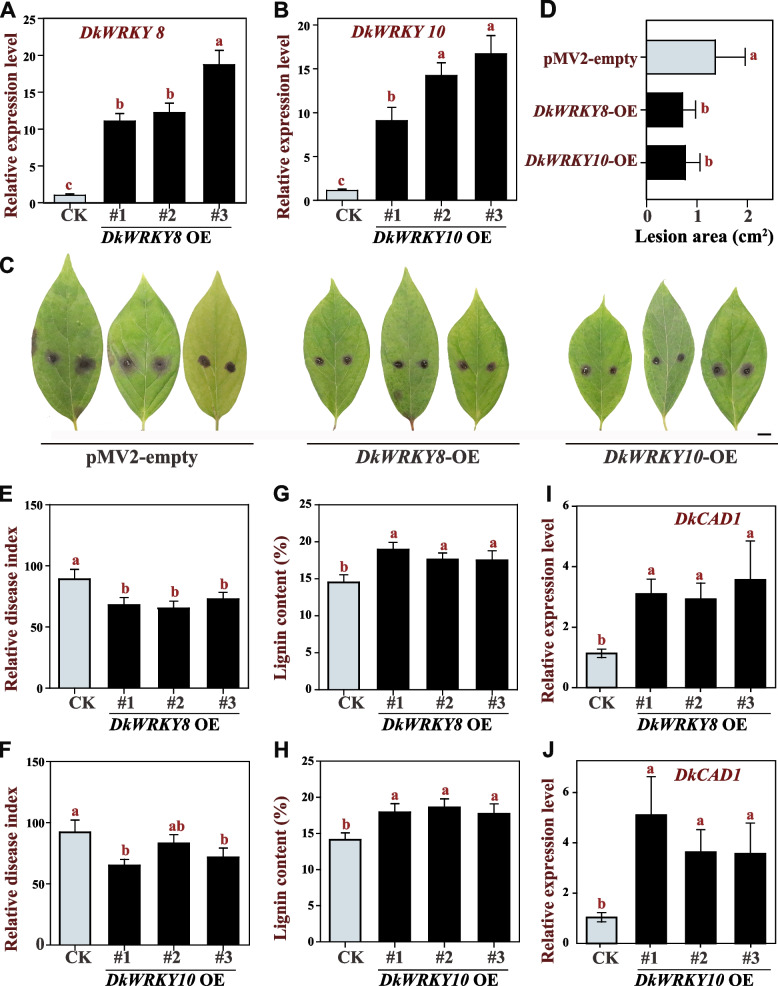
Enhanced resistance to *C. horii* in persimmon through *DkWRKY8* and *DkWRKY10* overexpression. **A**, **B** The expression level of *DkWRKY8* and *DkWRKY10* in persimmon leaves after transient overexpression. **C** Disease resistance of pMV-empty, *DkWRKY8*, and *DkWRKY10*-overexpressing leaves. Bars = 1 cm. **D** Quantification of the data shown in (**C**). **E**, **F** Relative disease index in the *DkWRKY8* and *DkWRKY10* overexpressing leaves. ‘Fuping Jianshi’ leaves infiltrated with *DkWRKY8* and *DkWRKY10* and collected leaves after eight days of agroinfiltration. OE1, OE2, and OE3 represent three different lines infiltrated with pMV2-*DkWRKY8* and pMV2-*DkWRKY10* vectors. The pMV2-GFP was used as a control. **G**, **H** Lignin content in the *DkWRKY8* and *DkWRKY10* overexpressing leaves. Error bars indicate the standard deviation (*n*=3). **I**, **J** The expression level of *DkCAD1* in transiently overexpressed lines of *DkWRKY8* and *DkWRKY10* in persimmon leaves. The letters indicate significant differences according to one-way ANOVA (Tukey’s test; *p* < 0.05)

To gain a deeper understanding of the roles played by *DkWRKY8* and *DkWRKY10* in the defense response against pathogens, we generated transgenic persimmon leaves overexpressing these genes using an *Agrobacterium*-mediated transient transformation system and vacuum infiltration. The overexpressed lines, namely OE1, OE2, and OE3 for both *DkWRKY8* and *DkWRKY10* were obtained, we then conducted a qRT-PCR analysis to assess the transcriptional abundance of *DkWRKY8* and *DkWRKY10*, and the results indicated that these two genes exhibited a significant increase across all three overexpressed lines, with expression levels 12-fold and 14-fold higher, respectively, compared with the infiltrated control leaves (Fig. 7A, B). Subsequently, the overexpressed lines of *DkWRKY8* and *DkWRKY10* were inoculated with *C. horii*. After 5 dpi, we observed a significant reduction in the average lesion area in leaves overexpressing *DkWRKY8* and *DkWRKY10* compared with the control group (Fig. 7C, D). Furthermore, there was a notable decrease in the disease index of plants overexpressing *DkWRKY8* and *DkWRKY10* (Fig. 7E, F), accompanied by a marked enhancement in lignin accumulation in their leaves (Fig. 7G, H). Additionally, we detected the expression levels of *DkCAD1* in *DkWRKY8* and *DkWRKY10* overexpression persimmon leaves, which suggested a positive regulation of *DkWRKY8* and *DkWRKY10* to the expression of *DkCAD1* (Fig. 7I, J). These findings indicated that, similar to *DkCAD1*, *DkWRKY8* and *DkWRKY10* play positive regulatory roles in modulating *C. horii* resistance in persimmon.

### Positive roles of* DkCAD1*,* DkWRKY8, *and* DkWRKY10* in *C. horii* resistance modulated by SA

**Fig. 8:**
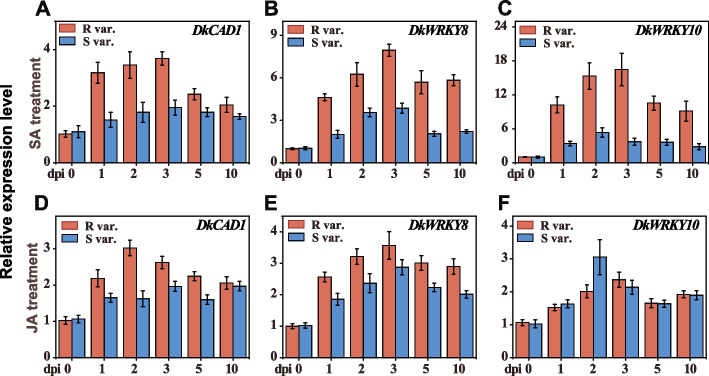
Positive roles of *DkCAD1*, *DkWRKY8*, and *DkWRKY10* in *C. horii* resistance modulated by SA. **A**-**C** Expression levels of *DkCAD1, DkWRKY8* and *DkWRKY10* in two persimmon cultivars inoculated with *C. horii* after 2 days of spraying 0.1 mM SA. **D**-**F** Expression levels of *DkCAD1, DkWRKY8* and *DkWRKY10* in two persimmon cultivars inoculated with *C. horii* after 2 days of spraying 0.1 mM JA. Samples were collected at 0, 1-, 2-, 3-, 5-, and 10-days post-inoculation (dpi). Error bars indicate the standard deviation (*n*=3, *p* < 0.05)

To investigate the impact of exogenous SA and JA on *C. horii* resistance, we applied these hormones to the leaves of various persimmon cultivars before inoculation. The results showed that both hormones, particularly SA, significantly reduced the severity of the disease in persimmon, especially in R var. (Figure S4). These findings suggest that SA and JA have the potential to induce resistance to anthracnose in persimmon, with a more pronounced effect in R var. To determine which hormones could influence the expression of *DkCAD1*, *DkWRKY8* and *DkWRKY10* in response to pathogen infection, we measured expression levels in the leaves of R var. and S var. inoculated with *C. horii* at 2 days after spraying 0.1 mM SA or JA. A substantial increase in the expression levels of *DkCAD1*, *DkWRKY8* and *DkWRKY10* was observed in the resistant cultivar following SA treatment, whereas no significant difference was observed between the susceptible cultivar and the control (Fig. 8A–C). Exogenous JA treatment also induced the expression of these three genes in two different resistant persimmon cultivars, although the differences were not as pronounced as those following SA treatment (Fig. 8D–F). In conclusion, these findings suggest that *DkCAD1*, *DkWRKY8*, and *DkWRKY10* can be strongly and moderately induced by exogenous SA and JA, respectively.

## Discussion

*Colletotrichum* spp. comprises a group of fungi known for their key role as plant pathogens, capable of infecting various economically valuable crops (Dean et al. 2012). Among these fungi, *C. horii* is the primary causal agent responsible for persimmon anthracnose, a prevalent disease in China’s persimmon production regions. As the demand for persimmon continues to rise, this disease has emerged as a critical factor hindering industry growth. Previous research has indicated that *C. horii* primarily infects young shoots, leaves (particularly petioles and veins), and fruit during the spring season. Subsequently, the fungus overwinters on the diseased tissues in the form of mycelium or conidia, facilitating its spread through wind, rain, and favorable weather conditions, including high temperature and humidity (Xie et al. 2010; Hassan et al. 2018). In our previous field investigation, we observed that persimmon anthracnose primarily affected the new shoots of infected persimmon branches. Additionally, we found that a higher degree of lignification in branches was associated with a lower risk of infection. To further examine the impact of lignin on disease resistance in persimmon branches, we selected two cultivars for the present study: the susceptible cultivar ‘Fuping Jianshi’ denoted as S var., and the resistant cultivar ‘Kangbing Jianshi’ denoted as R var., based on their levels of anthracnose resistance, which were determined in our previous research (Guan et al. 2022). The symptom development in S var. following artificial inoculation was consistent with previous findings (Xie et al. 2010; Hassan et al. 2018; Deng et al. 2019). R var. exhibited minimal lesions and higher lignin content following inoculation in the field, whereas S var. displayed pronounced symptoms and lower lignin levels. Notably, the lignin deposition rate in R var. branches was significantly more rapid than that of S var., resulting in significantly greater lignin deposition during the same growth period. Based on these findings, we hypothesized that lignin plays a crucial role in conferring resistance to anthracnose in R var.

*CAD* genes exert marked effects on plant growth, development, and response to both abiotic and biotic stresses. In recent years, numerous studies have provided evidence for the involvement of *CAD* genes in a plant’s defense against pathogen infections. For instance, *AtCAD5* (*AtCAD_D*) plays crucial roles in lignin synthesis and contribute to the resistance of *Arabidopsis thaliana* against *Pseudomonas syringae* pv. tomato infection (Sibout et al. 2003; Tronchet et al. 2010). Furthermore, the transcriptional level of *TaCAD12* was found to be markedly higher in two sharp eyespot-resistant wheat lines compared with susceptible wheat lines, and it exhibited a significant increase following *Rhizoctonia cerealis* infection (Rong et al. 2016). The present study aimed to elucidate the divergent expression patterns of *DkCAD1* in susceptible and resistant persimmon cultivars infected by *C. horii*. The expression of *DkCAD1* in R var. significantly increased following infection, surpassing the levels observed in S var. during the same period (Fig. 5A). These findings indicate that *DkCAD1* actively responds to the *C. horii* infection in resistant persimmon cultivars, and the high lignin content and expression level of *DkCAD1* are associated with the specific persimmon response upon *C. horii* infection. Additionally, our investigation revealed that transiently overexpressing *DkCAD1* in the leaves of S var. post inoculation resulted in a notable decrease in disease index and an increase in lignin content (Fig. 5E, F). These findings suggest that the presence of *DkCAD1* potentially enhances the persimmon’s resistance to *C. horii*, and we hypothesized that this resistance is attributed to the facilitation of lignin accumulation.

The WRKY transcription factor exhibits the capability to coordinate with other genes, orchestrating various plant processes (Bakshi and Oelmüller 2014). For instance, JrWRKY21 enhances the transcriptional activity of JrPTI5L by binding to the W-box motif in the promoter region. This interaction indirectly stimulates the expression of the *JrPR5L* gene through the formation of the WRKY21–PTI5L protein complex, ultimately leading to enhanced resistance against *C. gloeosporioides* in walnut (Zhou et al. 2022). Additionally, PtrWRKY89 demonstrates the ability to bind to the promoters of two potential downstream target genes, *PtrWRKY18* and *PtrWRKY35*, which can be induced by SA in *Melampsora* rust-infected poplar leaves (Jiang et al. 2017). In our previous study, we conducted Y1H assays to screen two DkWRKY transcription factors, namely DkWRKY3 and DkWRKY15 (Guan et al. 2020a). Overexpressing these factors resulted in the up-regulation of acetaldehyde metabolism-related gene *DkPK1*’s expression, as well as the positive regulation of natural deastringency in C-PCNA persimmon. Furthermore, we identified two additional WRKY transcription factors, DkWRKY8 and DkWRKY10, which bind to the promoter of *DkCAD1* using the same method. We found that *DkWRKY8* and *DkWRKY10* expression patterns in a resistant cultivar postinoculation were similar to those of *DkCAD1* (Figs. 5A and 6A). Moreover, the transient overexpression of *DkWRKY8* and *DkWRKY10* positively regulated the defense against *C. horii* infection in persimmon (Fig. 7). The results of the multiple sequence analysis demonstrated a notable similarity between DkWRKY10 and AtWRKY54 and AtWRKY70. Previous research has identified AtWRKY54 and AtWRKY70 as positive regulators that facilitate the expression of *SARD1* and *CBP60g* during plant defense responses (Chen et al. 2021). Our investigation suggests that DkWRKY8 and DkWRKY10 may play a positive regulatory role in the defending against *C. horii* infection in persimmon.

## Conclusions

In conclusion, our study has revealed that the resistant cultivar ‘Kangbing Jianshi’ exhibits minimal susceptibility to *C. horii* infection, and this resistance is attributed to its significantly higher lignin content compared with the susceptible cultivar ‘Fuping Jianshi’, which displays pronounced disease symptoms. In a resistant cultivar, the expression of *DkCAD1* was observed to vary following infection. Using the *DkCAD1* promoter as bait, we identified *DkWRKY8* and *DkWRKY10*, both of which interacted with this promoter. *DkWRKY8*, *DkWRKY10*, and *DkCAD1* were all positively associated with disease resistance in persimmon. Furthermore, both *DkWRKY8* and *DkWRKY10* were highly induced by exogenous SA but not JA in the resistant cultivar. Although further investigation is warranted, these findings represent a significant advancement in comprehending the mechanism of resistance to *C. horii* in diverse persimmon varieties. Therefore, this study serves as a valuable reference for the identification of additional persimmon genes associated with disease resistance and the breeding of new resistant germplasm.

## Materials and methods

### Plant and fungal material

Based on the previous investigation of germplasm resources regarding persimmon anthracnose resistance, the highly susceptible cultivar ‘Fuping Jianshi’ (S var.) and the highly resistance cultivar ‘Kangbing Jianshi’ (R var.) were used in this study (Guan et al. 2022). These materials were cultured at the National Field Genebank for Persimmon, located in Yangling, Shaanxi, China (34°17´42.80´´N, 108°04´8.21´´E). Leaves from the S Var. with similar healthy growth conditions were collected for further study, and *C. horii* strain FJ-1 was isolated from the infected S Var. branches (Fan et al. 2022), then incubated on potato dextrose agar (PDA) at 25°C in the light incubator.

### Pathogen inoculation and hormone treatments

The conidial suspension for pathogen inoculation was obtained according to the previous descriptions (Hopkins and Harris 2000). The mycelia were grown on PDA plate for 7-10 days, after which they were scraped off using a scalpel and suspended in aseptic distilled water. The resulting conidial suspension was filtered through aseptic gauze and adjusted to 1×10^6^ conidia/ml. The conidial suspension was applied to the young shoots of persimmon plants, which were subsequently bagged to maintain humidity after inoculation. Infected leaves were collected at various time points (0, 1, 3, 5, 7, and 10 days) following inoculation, frozen in liquid nitrogen, and stored at -80℃ refrigerator. Three biological repetitions were set and each consisting of 3-5 branches (Guan et al. 2022).

For SA and JA treatment, the leaves from S Var. and R Var. were sprayed with 0.1 mM SA or JA. After two days, the leaves were exposed to *C. horii* for a duration of two weeks in an incubator with 70% humidity and 25–28°C.

### Endogenous contents of SA and JA

Endogenous SA and JA contents were determined using the previously described method (Fu et al. 2012). Briefly, 100 mg infected leaves were ground into powder and then placed in 2 ml methanol solution. The resulting homogenate was stored at -20℃ for 10 h and subsequently centrifuged at 12,000 g for 12 min at 4℃. The supernatant was dissolved in a 5% ammonia solution and purified using the Oasis MAX solid-phase extraction (SPE) column (Waters, Milford, MA). The eluted samples were then subjected to centrifuging at 12000 g for 5 min at 4℃, and the resulting supernatant was collected for the detection of SA and JA content with LC-MS (SCIEX, QTRAP5500).

### Lignin content measurement and histochemical staining

The lignin content of branches was quantified using the Klason method (Fagerstedt et al. 2015). Specifically, 0.5 g (W1) samples were subjected to extraction in a Soxhlet apparatus using a mixture of ethanol and benzene (volume ratio 1:2) for 6 h. Subsequently, the extracted samples were dried to achieve a constant weight (W2). The dried powders were then treated with 72% sulfuric acid for 2 h and diluted with distilled water until the sulfuric acid concentration reached 3.0%. Following boiling and refluxing for 4 h, an insoluble residue was obtained, which was subsequently filtered and washed until it reached a pH of approximately 7. Finally, the residue was dried and weighed (W3). These measured values were utilized in the calculation of the lignin content: Lignin content (%) = (W2-W3)/W1×100.

The total lignin content was measured using the phloroglucinol-HCl staining method (Blanco-Portales et al. 2002). Specifically, an ethanol solution with 1% phloroglucinol was applied to cross- branches section to remain for 1-2 min, followed by the addition of concentrated hydrochloric acid (HCl) solution. The stained samples were observed using a stereomicroscope after the coloration process.

### RNA extraction and cDNA synthesis

Total RNA was extracted from infected branches of the S var. and R var. varieties using the RNAprep Pure Plant Plus Kit (DP441, Tiangen Biotech Co., Beijing, China). Three biological replicates were included for each sample. The quality and concentration of the RNA were evaluated through gel electrophoresis analysis and spectrophotometer detection (NanoDrop 2000, Thermo Fisher Scientific, USA). For cDNA synthesis, the removal of genomic DNA and the synthesis of the first-strand cDNA were carried out using the PrimeScript^TM^ RT Reagent Kit with gDNA Eraser according to the manufacturer’s instructions (TaKaRa, Dalian, China).

### Transcriptome analysis

The uninfected (CK), infected branch samples from S var. and R var. varieties were collected for sequencing at 1, 3, 5 dpi. The samples were then sent to Genedenovo Biotechnology Co., Ltd (Guangzhou, China) for library construction, and subsequent sequencing was conducted using Illumina HiSeqTM4000. Raw sequencing data were preprocessed with fastp and Kallisto was used for read counts qualification of each transcript and ‘Xiaoguotianshi’ was used as the reference genome (Li et al. 2023). Furthermore, basic functional annotation and difference gene analysis were performed using Gene Ontology, KEGG, and Mapman annotation (Thimm et al. 2004). DESeq2 was employed to analyze the differential expression genes between CK and 1,3,5 dpi samples. The identification of significant genes was based on a *p*-value threshold of less than 0.01 or a log2 fold change greater than 1. Gene expression value was represented by transcripts per million values (TPM) and normalized using a log2 (TPM +1) transformation with DESeq2. K-means clustering method was implemented for the differential genes using the hk-means function in the R package ‘factoextra’, with K set to 12.

### Metabolomics analysis

Non-targeted metabolomics analysis was performed with the infected branches from S var. and R var. Each group consisted of six biological replicates. The metabolites were extracted and measured using a Liquid chromatograph-mass spectrometer (LC-MS). Agilent 1290 ultra-high-performance liquid chromatography and Thermo Fisher Scientific Q Exactive Orbitrap mass spectrometer was used in tandem for analysis. A standard and advanced analysis of the samples was conducted following detection. To identify differentially accumulated metabolites (DAMs), the screening condition was set as variable importance in the projection (VIP) ≥ 1 and T-test *P* < 0.05. Moreover, the relationship between genes and metabolites was examined. The average log2 value was calculated from the biological replicates, and the Pearson correlation coefficient was then calculated for the resulting data.

### Quantitative real-time analysis

Quantitative real-time polymerase chain reactions (qRT-PCR) was conducted using the ABI One Step Plus Real-Time PCR System (Applied Biosystems, Carlsbad, CA, USA). The PCR reaction mixture consisted of a total volume of 20 µl, including 10 µl SYBR Premix Ex Taq II (TaKaRa, Dalian, China), 7.4 µl ddH_2_O, 1.0 µl diluted cDNA, and 0.8 µl of each primer (10 µM). The PCR conditions were as follows: an initial denaturation step at 95°C for 5 min, followed by 45 cycles of denaturation at 95 °C for 5 s, annealing at 58°C for 10 s, and extension at 72°C for 15 s. *DkActin* was utilized as an internal reference (Akagi et al. 2009), and data analysis was performed using the 2^–ΔΔ^Ct method (Livak and Schmittgen 2001). The primer pairs used in this study were listed in Table S8.

### Dual-luciferase assay

The dual-luciferase assay was employed to investigate the transcriptional activation of *DkCAD1* by DkWRKY (Hellens et al. 2005). Following the methodology described previously (Min et al. 2012), the pGreen II 002962-SK (SK) vector was used to insert the full-length sequences of *DkWRKY8* and *DkWRKY10* while the promoter fragments of *DkCAD1* were fused into the pGreen II 0800-LUC (LUC) vectors. These constructs were then transformed into *Agrobacterium tumefaciens* strains GV3101 and transiently expressed in *Nicotiana benthamiana* leaves. After three days, the LUC and REN contents were analyzed using a dual-luciferase reporter assay system (Promega, USA). Three biological replicates were conducted, and each contained three technical replicates.

### Yeast one-hybrid analysis

Yeast one-hybrid analysis was used to validate the interactions between DkWRKY8, DkWRKY10, and the promoter of *DkCAD1* according to the Matchmaker™ Gold Yeast One-Hybrid Library Screening System (Clontech, USA). The *DkCAD1* promoter was inserted into the pAbAi vector, and BstBI was used for digesting the recombinant plasmids. The linearized recombinant plasmids were transformed into the yeast strain Y1H and tested for Aureobasidin A (AbA) concentrations (100−500 ng/mL) on SD/-Ura medium. The coding sequence of *DkWRKY8* and *DkWRKY10* were cloned into the pGADT7 vectors and transferred into the competent cells with recombinant plasmids of promoter of *DkCAD1*. The transformants were cultured on SD/-Leu medium with supplemented with 100 and 200 ng/ml AbA, respectively. pGADT7 (AD-p53) and p53-AbAi were employed as positive controls.

### Transient transformation of *DkCAD1* in persimmon leaves

To ascertain the functional role of *DkCAD1*, *DkWRKY8*, and *DkWRKY10* during pathogen infection, the overexpression vector pMV2-*DkCAD1*, pMV2-*DkWRKY8*, and pMV2-*DkWRKY10* were constructed and transiently expressed into the S Var. leaves by vacuum infiltration (Mo et al. 2019), pMV2-GFP construct was used as control. The strain *C. horii* strain FJ-1 was inoculated into the overexpressed leaves for a duration of 2 days. Subsequently, images were captured at 5 days post-inoculation (dpi) to assess the relative resistance index (Guan et al. 2022), and the lignin content was determined using the Klason method (Fagerstedt et al. 2015). Six sets were conducted, each set comprising three biological replicates and each replicate consisted of a minimum of 10 leaves.

### Statistical analysis

The obtained data were subjected to statistical analysis using one-way analysis of variance (AVNOA) with Duncan’s multiple range test using SPSS 22.0 software (IBM SPSS Statistics, Chicago, IL, USA). Statistical significance was considered at *p* < 0.05.

### Supplementary Information

**Additional file 1: Table S1.** Normalized metabolic profile of DAMs.

**Additional file 2:** **Table S2.** Matrix of -log10 *p*-value from GO annotation enrichment analysis of DEGs.

**Additional file 3:** **Table S3.** Matrix of -log10 *p*-value from KEGG annotation enrichment analysis of DEGs.

**Additional file 4:** **Table S4.** Matrix of -log10 *p*-value from Mapman annotation enrichment analysis of DEGs.

**Additional file 5:** **Table S5.** Normalized expression value of JA and SA related genes from RNA-seq data.

**Additional file 6:** **Table S6.** Expression value of lignin synthesis related genes from RNA-seq data.

**Additional file 7:** **Table S7.** Gene expression value of CAD family from RNA-seq data.

**Additional file 8:** **Table S8.** Primers used in this study.

** Additional file 9: Figure S1.** GO annotation enrichment analysis of DEGs. **Figure S2.** Normalized profile of SA and JA related DEGs in S var. (A) and R var. **Figure S3.** Promoter sequence of DkCAD1. **Figure S4.** Effects of exogenous SA and JA on C. horii resistance.

## Data Availability

All data generated or analyzed during this study are included in this published article or on its supplementary information.

## Abbreviations

*C. horri*: *Colletotrichum horii*
R var.: ‘Kangbing Jianshi’ cultivar
S var.: ‘Fuping Jianshi’ cultivar
Y1H: Yeast one-Hybrid
NFGP: National Field Genebank for Persimmon
CAD: Cinnamyl Alcohol Dehydrogenase
PDA: Potato Dextrose Agar
SA: Salicylic Acid
JA: Jasmonic Acid
ROS: Reactive Oxygen Species
TFs: Transcription factor
DEG: Differentially Expressed Genes
DAM: Differentially Accumulated Metabolites
GO: Gene Ontology
KEGG: Kyoto Encyclopedia of Genes and Genomes
SPE: Solid-Phase Extraction
TPM: Transcripts Per Million
LC-MS: Liquid Chromatography-Mass Spectrometry
qRT-PCR: Quantitative real-time polymerase chain reactions
AbA: Aureobasidin A
dpi: days post-inoculation
C-PCNA: Chinese pollination-constant non-astringent
