# Supplementary material for: DkWRKY transcription factors enhance persimmon resistance to Colletotrichum horii by promoting lignin accumulation through DkCAD1 promotor interaction
Source: Stress Biol. 2024 Feb 26;4(1):17. doi: 10.1007/s44154-024-00154-0 (PMC10897097; doi:10.1007/s44154-024-00154-0)
Supplement: Supplementary file 9 — Additional file 9: Figure S1. GO annotation enrichment analysis of DEGs. Figure S2. Normalized profile of SA and JA related DEGs in S var. (A) and R var. Figure S3. Promoter sequence of DkCAD1. Figure S4. Effects of exogenous SA and JA on C. horii resistance. [file 44154_2024_154_MOESM9_ESM.pdf]

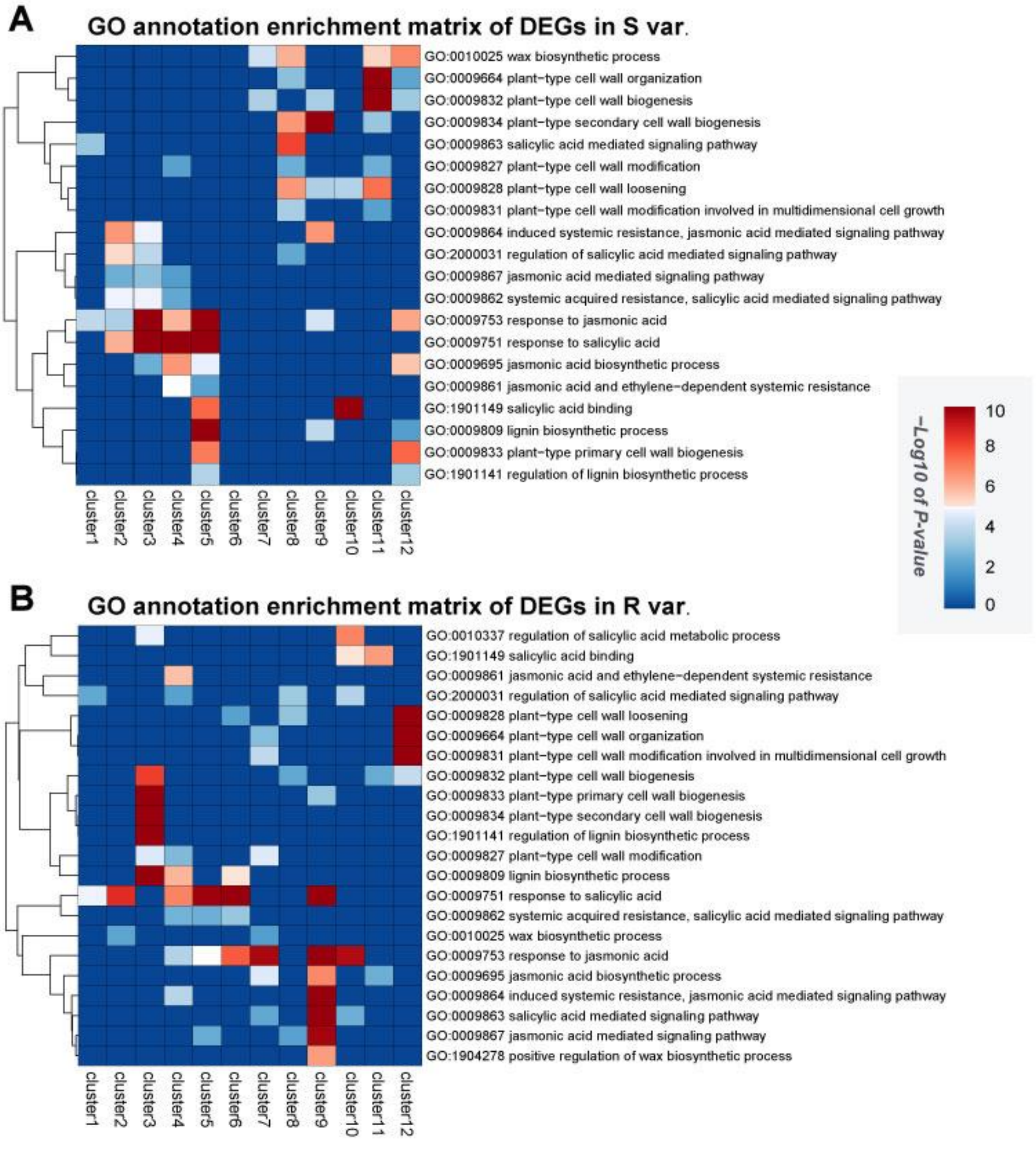

Figure S1. GO annotation enrichment analysis of DEGs. R var. represents ‘Kangbing Jianshi’, and S var. represents ‘Fuping Jianshi’. DEG, differentially expressed genes.

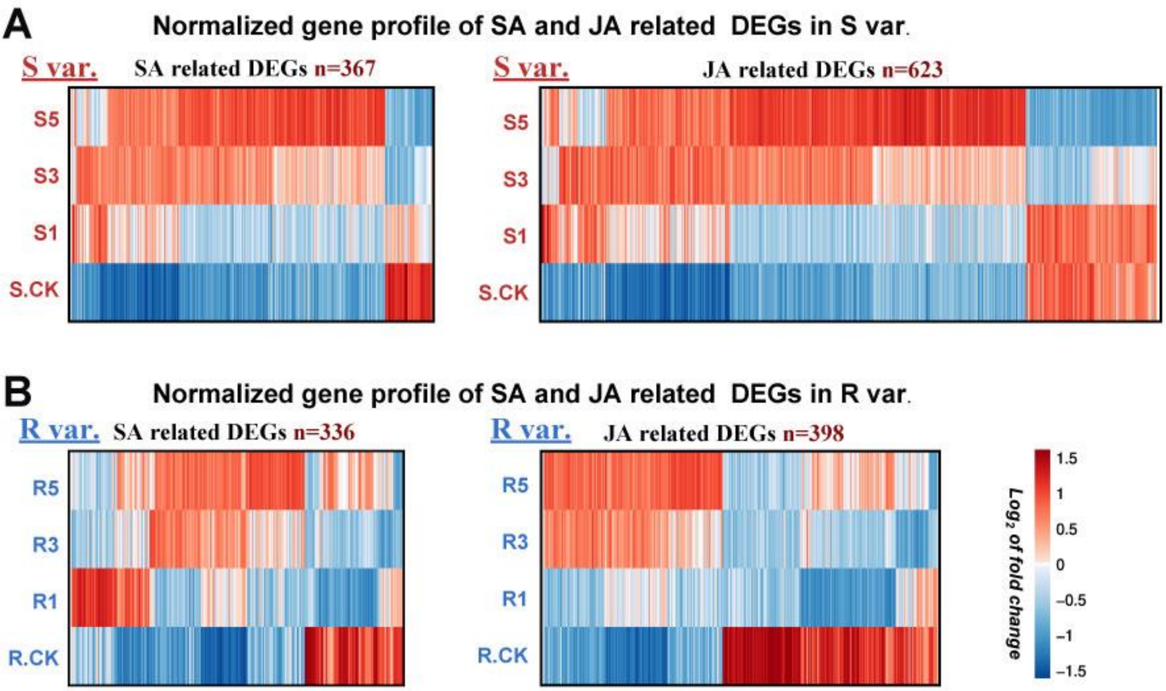

Figure S2. Normalized profile of SA and JA related DEGs in S var. (A) and R var. (B). R var. represents 'Kangbing Jianshi', and S var. represents 'Fuping Jianshi'. 1, 3, and 5 represent the time points for collecting samples. DEG, differentially expressed genes.

>Promoter of *DkCAD1*

(F)CACATCCAAACCACCCATCACAATTAGCTTTCCCGATTCACGCTGG
CACTCAAGTTTTAGTTAATCATGCAAACTAGGCATAAGCTCCATGATC
AGCTCAGAGAATTTGTAACTGTGTGAGAGGGGGAGGGCCATGAATTT
GCAAATATATTTAGATGAAGGGAGCATTCACAGGCTTTAACCAATCA
CTTTCAATCCCATAATCATATTTTTAAAAAATCCTATTATTAAACCAC
GTAATGATAAAAAATATATATTTATGAATTTATTATCGTCAAACAATA
ATTGAATATTTGTGTTCATAATCGTGGTGTAAATGTTGGGGAAAAAA
AATATCTAAGAAGGCTAAGGCATGCCAGTTTGAGAGAATGTTTATGG
GAATACATTTGATTTTGAGTCTTGAATTAACTTAATTTGAATAAAAAA
AAGTAAAAAAAAATTGAAATAGTTAAAATACGCAAATTCTCTTGAAA
CAAACTGAACTATCGTAGAAGTTTTGTTTTTTTTTAAAAAAAAATTCT
TTTAGTATAATCAGCATTTACAACACTTGTTGTCTTAAACTGAAGGAA
TAGCAAACTGTTAAATTCCAAAAAAATAAAATAAAAAACAAAAAAA
CTTTTAAATATTAGCCAAGTAAAATATAGTAAGTTGAAAATGACTGA
CTGGAATATGATGTTAGCCGTGACGCCTTGGAGAGACGTGCCTATCA
GGAAAACCTCCGGCGGCTGAGATACGTGTCTAACACCAGCACCTCCG
GCGCGGGGGCGGACATATTTATTTATATGCATCTGAGAGGGGAGAAG
AA (R)GAGTAAGGAGCAGAGCAGAGATG

Figure S3. Promoter sequence of *DkCAD1*. Marked in red are the sequences of primers F and R.

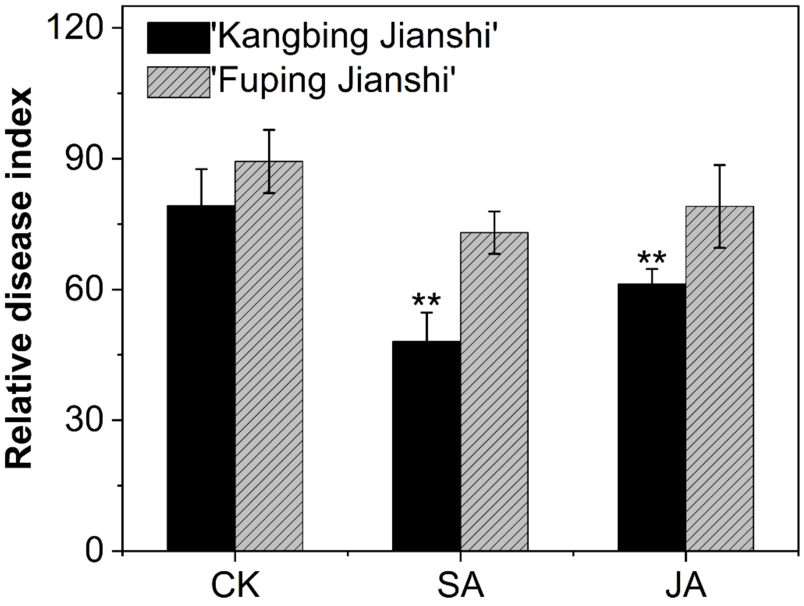

Figure S4. Effects of exogenous SA and JA on *C. horii* resistance. ‘Fuping Jianshi’ and ‘Kangbing Jianshi’ leaves were inoculated with *C. horii* at 2 days after spraying 0.1 mM SA or JA then the leaves were collected for total RNA extraction and qRT-PCR analysis. Error bars indicate the standard deviation (n=3, $p < 0.05$).
